# A combined eGFR-CD4 index and mortality risk among people with HIV: a retrospective cohort study in Taizhou city, Zhejiang province, China

**DOI:** 10.1186/s12981-026-00894-1

**Published:** 2026-06-07

**Authors:** Congcong Guo, Xinxin Xing, Qiguo Meng, Shanling Wang, Yali Xie, Tailin Chen, Jiyuan Ren, Xiaoxiao Chen, Haijiang Lin

**Affiliations:** 1 Jiaojiang Center for Disease Control and Prevention (Jiaojiang Institute of Health Supervision), Taizhou, 318000 Zhejiang China; 2 Taizhou Center for Disease Control and Prevention (Taizhou Institute of Health Supervision), Taizhou, 318000 Zhejiang China; 3 Department of Epidemiology, School of Public Health, Fudan University, Shanghai, 200032 China; 4 Taizhou Central Blood Station, Taizhou, 318000 Zhejiang China

**Keywords:** HIV, Mortality, CD4, Estimated glomerular filtration rate, Risk stratification

## Abstract

**Background:**

Mortality among people with HIV (PWH) increasingly reflects both HIV-related and non-HIV-related causes. Whether a simple index integrating routinely measured kidney and immune function can improve risk stratification remains uncertain.

**Methods:**

We conducted a retrospective cohort study in Taizhou, Zhejiang, China using the China HIV/AIDS Comprehensive Response Information Management System linked to a cause-of-death surveillance system. Adults aged ≥ 18 years diagnosed with HIV between 1998 and 2024 who had at least one paired serum creatinine and CD4 measurement were followed through 31 December 2024. Time-updated estimated glomerular filtration rate (eGFR), CD4, and a combined eGFR-CD4 index (CBI) were analysed using time-dependent Cox models. Restricted cubic splines assessed non-linearity, and 12-month landmark analyses with time-dependent receiver operating characteristic curves evaluated discrimination. Bootstrap internal validation was performed.

**Results:**

Among 3683 PWH (median follow-up 5.61 years), 434 deaths occurred, including 67 HIV-related and 367 non-HIV-related deaths. Each 1-SD increase in CBI was associated with higher risks of all-cause, HIV-related, and non-HIV-related mortality, with adjusted hazard ratios of 4.12 (95% CI: 2.76–6.15), 37.86 (95% CI: 9.03–158.80), and 3.30 (95% CI: 2.20–4.93), respectively (all *P* < 0.001). Landmark analyses showed graded risk separation across CBI quartiles. After bootstrap internal validation, the combined model showed modestly higher discrimination for all-cause and non-HIV-related mortality, whereas CD4 alone performed best for HIV-related mortality.

**Conclusions:**

A time-varying combined eGFR-CD4 index was associated with mortality outcomes in this cohort of PWH in Taizhou, Zhejiang, China. Its discriminatory advantage after internal validation was modest and was evident for all-cause and non-HIV-related, but not HIV-related, mortality. External validation is warranted.

**Supplementary Information:**

The online version contains supplementary material available at 10.1186/s12981-026-00894-1.

## Background

People with HIV (PWH) are living longer in the antiretroviral therapy (ART) era, yet mortality remains substantial and increasingly reflects a mix of HIV-related and non-HIV-related causes. In 2024, an estimated 40.8 million (37.0–45.6 million) people were living with HIV worldwide, and 630,000 (490,000–820,000) died from HIV-related causes [1, 2]. As ART coverage expands and populations age, the proportion of deaths attributable to non-AIDS-related conditions has increased in many settings [3, 4]. In China, national surveillance likewise suggests changing cause-of-death patterns alongside the scale-up of universal ART policies [5].

Two routinely collected clinical markers capture key biological domains central to long-term prognosis in PWH: kidney function and immune status. Reduced estimated glomerular filtration rate (eGFR) reflects kidney impairment and cumulative comorbidity burden and has been associated with higher mortality risk in diverse HIV cohorts [6–9]. CD4 cell count remains a cornerstone marker of immune function; longitudinal studies demonstrate a strong gradient between time-updated CD4 levels and subsequent risk of AIDS events and death, with evidence that risk reductions may plateau at higher CD4 ranges [10, 11].

Multivariable risk scores, such as the Veterans Aging Cohort Study (VACS) Index 2.0, integrate HIV-related and general biomarkers and show good discrimination for mortality [12]. However, their complexity may limit routine implementation, and they are not designed to directly address HIV-related vs. non-HIV-related mortality in settings where causes of death are shifting.

Whether contemporaneous renal and immune function jointly contribute to prognosis when modeled dynamically over time—and whether a simple combined eGFR-CD4 index improves risk stratification for both HIV-related and non-HIV-related mortality—remains insufficiently characterized in real-world cohorts from China. In this retrospective cohort study of PWH in Taizhou, Zhejiang, China, we evaluated the time-varying associations of eGFR, CD4, and a combined eGFR-CD4 index with all-cause mortality, HIV-related mortality, and non-HIV-related mortality, and assessed whether the combined index improved risk stratification and discrimination compared with individual biomarkers.

## Methods

### Research design and study population

This retrospective cohort study was conducted in Taizhou city, Zhejiang province, China. Individual-level data were obtained from the China HIV/AIDS Comprehensive Response Information Management System (CRIMS) and linked to the Taizhou Cause-of-death monitoring system using unique identifiers [13]. Death status and cause-of-death information were obtained through this record linkage. HIV-related deaths were classified using surveillance records together with the underlying cause-of-death ICD-10 codes to capture AIDS and AIDS-defining conditions, whereas all remaining deaths were classified as non-HIV-related.

**Fig. 1:**
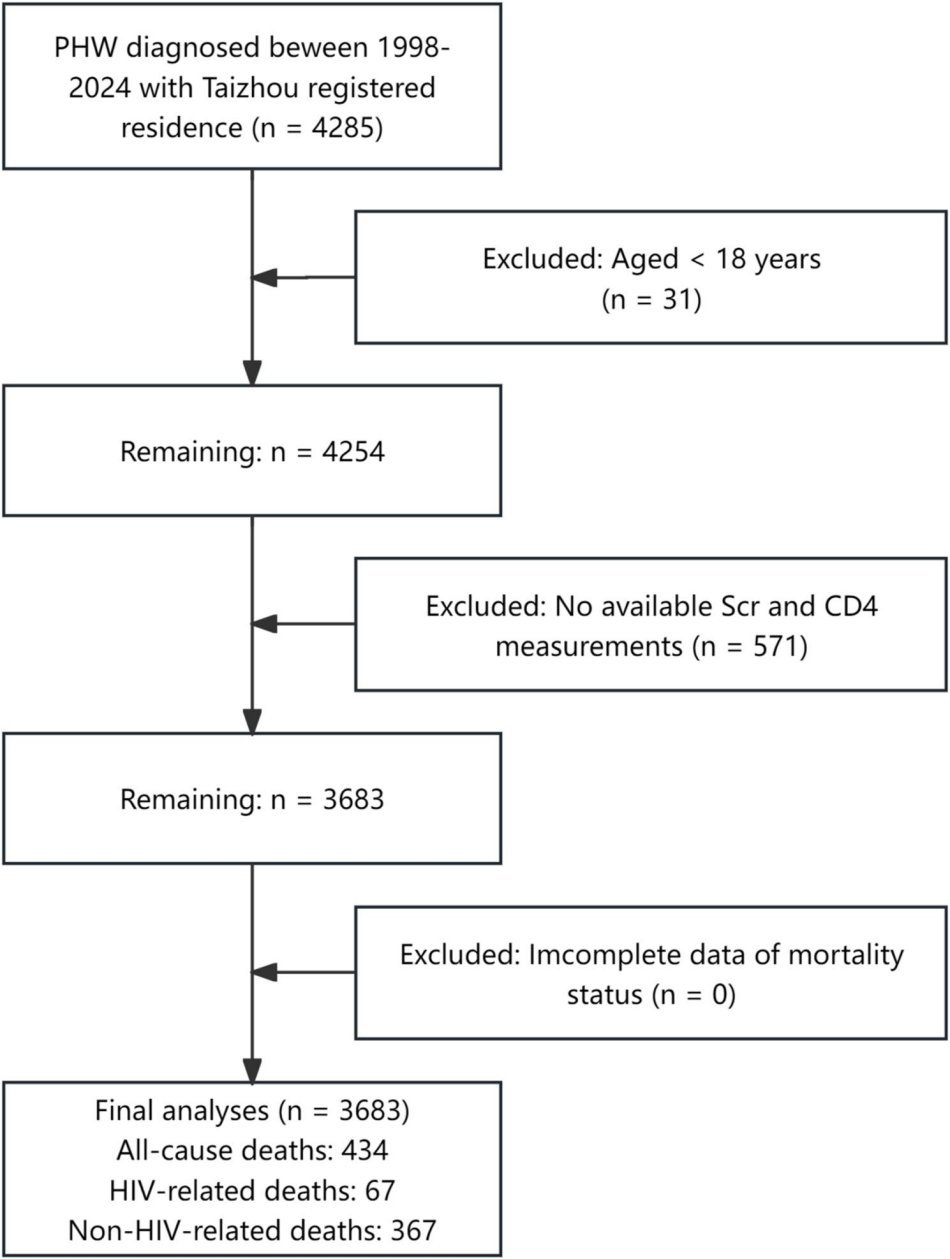
Flowchart for participants selected

We initially identified 4285 PWH who were diagnosed between 1998 and 2024 and had registered residence in Taizhou. Individuals aged < 18 years at the time of the first available serum creatinine (Scr) and CD4 measurement were excluded (*n* = 31). We further excluded participants without Scr and CD4 measurements (*n* = 571). Cohort inclusion therefore required at least one paired Scr and CD4 measurement, which may have preferentially selected individuals with more complete clinical monitoring and follow-up. The final analytic cohort comprised 3683 PWH, among whom 434 died during follow-up, including 67 HIV-related deaths and 367 non-HIV-related deaths (Fig. 1).

### Time-varying biomarkers and combined eGFR-CD4 index

Scr was used to calculate estimated glomerular filtration rate (eGFR) using the 2021 race-free CKD-EPI creatinine equation [14]. eGFR (mL/min/1.73 m^2^) and CD4 cell count (cells/µL) were modeled as time-varying covariates. Time zero (baseline) was defined as the date of the first paired eGFR and CD4 measurement, and follow-up accrued until death or censoring. In the routine surveillance and clinical care setting, Scr and CD4 were treated as paired observations for longitudinal analysis, although laboratory testing or reporting could occur on different dates. Repeated paired eGFR and CD4 observations were incorporated into a start-stop counting-process dataset, with biomarker values updated at each new paired observation and carried forward until the next available paired observation, death, or censoring (last observation carried forward).

A combined eGFR-CD4 index (CBI) was constructed as a linear combination of time-updated eGFR and CD4 using coefficients from the fully adjusted time-dependent Cox model. The coefficients used to define the CBI were estimated in the same analytic cohort in which the index was subsequently evaluated.

$${\text{CBI(t) = ~ - 0}}{\mathrm{.004062488}} \cdot {\text{eGFR(t) + - 0}}{\mathrm{.004197109}} \cdot {\mathrm{CD4(t)}}$$

Higher CBI values correspond to lower eGFR and lower CD4, indicating poorer renal-immune status.

### Outcomes

The primary outcome was all-cause mortality. Cause-specific outcomes were HIV-related and non-HIV-related mortality, defined using the underlying cause of death from the surveillance system. Cause-specific analyses used a cause-specific hazard framework, in which deaths from other causes were treated as censoring events at the time of death. Participants who were lost to follow-up were censored at the date of their last recorded follow-up. Cumulative incidence functions (CIFs) were also estimated using the Aalen-Johansen method accounting for competing risks [15].

### Time-dependent Cox regression

Associations of time-updated eGFR, time-updated CD4, and time-updated CBI with mortality were assessed using time-dependent Cox proportional hazards models [16]. Robust variance estimates were computed using clustering at the participant level (cluster[ptid]) to account for within-participant correlation. A prespecified adjustment strategy was used: Model 1 included the exposure of interest only; Model 2 additionally adjusted for demographic and socioeconomic characteristics (sex, age, occupational status, education level, marital status, and BMI); and Model 3 further adjusted for HIV-related factors including disease stage, diagnostic delay, transmission route, initial antiretroviral regimen, and time from diagnosis to ART initiation. Covariates had low levels of missingness and were handled using complete-case analysis in the fully adjusted models. Associations for CBI were reported per 1-unit increase (original scale) and per 1-SD increase (standardized scale). Sensitivity analyses included alternative scaling (per IQR), restriction to the 1st–99th percentiles, and exclusion of deaths within 12 months of follow-up (Supplementary Table S1). Hazard ratios (HRs) and 95% confidence intervals (CIs) were reported. Stratified analyses were performed to explore potential heterogeneity of associations.

### Kaplan–Meier curves

To visualize risk stratification by CBI, Kaplan-Meier curves were generated using a landmark approach. The landmark time was prespecified as 12 months after the first measurement. Participants alive and under follow-up at the landmark were included and grouped into quartiles (Q1–Q4) based on the first-year time-weighted mean CBI. The first-year time-weighted mean eGFR, CD4, and CBI were calculated from the paired time-updated observations accrued between baseline and the 12-month landmark. Survival time was recalculated from the landmark date to death or censoring. Differences across quartiles were assessed using the log-rank test, and numbers at risk were displayed.

### Time-dependent ROC curves

Time-dependent receiver operating characteristic (ROC) curves were used to compare the discrimination of eGFR, CD4, and their combined model within the 12-month landmark framework. For each participant, first-year time-weighted mean eGFR and CD4 were calculated. From the 12-month landmark onward, Cox models were fitted to generate risk scores for 3 models (eGFR only, CD4 only, and combined). ROC curves and AUC at 3 and 5 years after the landmark were estimated using the *timeROC* method with marginal weighting [17]. For HIV-related and non-HIV-related mortality, we evaluated cause-specific discrimination by coding the event of interest as 1 and treating deaths from other causes as censored at the time of death.

We performed bootstrap internal validation within the 12-month landmark framework to assess potential optimism in model discrimination. For all-cause, HIV-related, and non-HIV-related mortality, Cox models were fitted for eGFR alone, CD4 alone, and the combined model, and AUCs were estimated at 3 and 5 years after the landmark. Using 200 bootstrap resamples at the participant level, each model was refitted in the bootstrap sample and then evaluated both in the bootstrap sample and in the original dataset. Optimism was calculated as the difference between apparent and test AUCs, and optimism-corrected AUCs were obtained by subtracting the mean optimism from the apparent AUC in the original dataset. For cause-specific outcomes, deaths from other causes were treated as censoring events.

### Restricted cubic spline analysis

To assess potential nonlinearity, restricted cubic splines (RCS, 4 knots) for time-updated eGFR, CD4, or CBI were fitted within time-dependent Cox models for each outcome and adjusted for the covariates in Model 3 [18]. Robust standard errors were computed with cluster(ptid). HRs were centered at the biomarker median (HR = 1) and plotted on a log10 scale within the 1st-99th percentiles. Overall and nonlinear effects were assessed using Wald tests.

Additional methodological details are provided in the Supplementary Methods. We used the STROBE reporting guideline to draft this manuscript, and the STROBE reporting checklist when editing, included in Supplementary Table S1 [19].

## Results

### Participant characteristics

A total of 3683 participants were included. Over a median follow-up of 5.61 years, 3237 participants remained alive, 12 were lost to follow-up, and 434 all-cause deaths were recorded, including 67 HIV-related deaths. Compared with participants who died, those who were alive or censored were younger and more likely to have stable employment, be married, and have higher education attainment and BMI; they also had a higher proportion of HIV disease stage (vs. AIDS) and male-to-male sexual transmission as the transmission route (all *P* < 0.05). Baseline CD4 count and the time-weighted mean CD4 during the first year were highest among participants who were alive/censored and lowest among those who died of HIV-related causes (both *P* < 0.001). The first-year time-weighted mean eGFR was also highest in the alive/censored group (*P* < 0.001). In contrast, the combined eGFR-CD4 index was lowest in the alive/censored group and highest in HIV-related deaths at both baseline (alive/censored: −1.56 (−2.16, −1.08) vs. HIV-related death: −0.78 (−1.23, −0.56)) and as the first-year time-weighted mean (alive/censored: −1.72 (−2.32, −1.20) vs. HIV-related death: −1.07 (−1.55, −0.76)) (both *P* < 0.001) (Table 1).

**Table 1:** Baseline and landmark characteristics of patients by mortality status^*1*^

Variable	Overall (*n* = 3683)	Alive/censored (*n* = 3249)	HIV-related deaths (*n* = 67)	Non-HIV-related deaths (*n* = 367)	*P*-value^2^
Section A. Baseline characteristics at cohort entry					
Age (years)	49.00 (34.00, 61.00)	48.00 (33.00, 59.00)	57.00 (39.00, 71.00)	62.00 (50.00, 71.00)	< 0.001
Sex					< 0.001
Male	3024 (82.1)	2639 (81.2)	52 (77.6)	333 (90.7)	
Female	659 (17.9)	610 (18.8)	15 (22.4)	34 (9.3)	
Occupational status					0.039
Stable employment	136 (3.7)	129 (4.0)	2 (3.0)	5 (1.4)	
Unstable or manual employment	2808 (76.2)	2456 (75.6)	48 (71.6)	304 (82.8)	
High-mobility/vulnerable	680 (18.5)	611 (18.8)	16 (23.9)	53 (14.4)	
Unknown	59 (1.6)	53 (1.6)	1 (1.5)	5 (1.4)	
Education level					< 0.001
Primary school or less	1492 (40.5)	1225 (37.7)	37 (55.2)	230 (62.7)	
Middle school	1162 (31.6)	1046 (32.2)	24 (35.8)	92 (25.1)	
High school or above	1029 (27.9)	978 (30.1)	6 (9.0)	45 (12.3)	
Marital status					< 0.001
Married	1893 (51.4)	1685 (51.9)	28 (41.8)	180 (49.2)	
Unmarried	951 (25.8)	886 (27.3)	18 (26.9)	47 (12.8)	
Divorced/Widowed	837 (22.7)	677 (20.8)	21 (31.3)	139 (38.0)	
BMI (kg/m ^2^ )	21.80 (19.92, 23.94)	21.98 (20.07, 24.06)	20.16 (18.03, 22.04)	20.57 (19.03, 22.49)	< 0.001
Disease stage					< 0.001
HIV	1833 (49.8)	1709 (52.6)	8 (11.9)	116 (31.6)	
AIDS	1850 (50.2)	1540 (47.4)	59 (88.1)	251 (68.4)	
Diagnostic delay (months)					0.011
<3	3316 (90.0)	2931 (90.2)	54 (80.6)	331 (90.2)	
3–12	120 (3.3)	97 (3.0)	5 (7.5)	189 (4.9)	
≥12	247 (6.7)	221 (6.8)	8 (11.9)	18 (4.9)	
Transmission route					< 0.001
Male-to-male sexual contact	1054 (28.7)	996 (30.7)	8 (11.9)	50 (13.6)	
Other routes	2624 (71.3)	2248 (69.3)	59 (88.1)	317 (86.4)	
Initial ART regimen					0.106
Non-first-line	388 (10.5)	342 (10.5)	12 (17.9)	34 (9.3)	
First-line	3294 (89.5)	2906 (89.5)	55 (82.1)	333 (90.7)	
Time to ART initiation (months)					0.633
<1	2859 (77.7)	2530 (77.9)	54 (80.6)	275 (74.9)	
1–3	288 (7.8)	248 (7.6)	5 (7.5)	35 (9.5)	
≥3	534 (14.5)	469 (14.4)	8 (11.9)	57 (15.5)	
Baseline eGFR (mL/min/1.73 m^2^)	106.63 (96.45, 118.21)	107.38 (97.69, 118.73)	107.62 (88.46, 120.54)	98.92 (88.61, 110.98)	< 0.001
Baseline CD4 (cells/µL)	260.00 (139.00, 394.50)	269.82 (153.00, 405.05)	84.60 (21.00, 187.00)	186.00 (72.00, 319.00)	< 0.001
Baseline combined eGFR-CD4 index	−1.52 (−2.11, −1.00)	−1.56 (−2.16, −1.08)	−0.78 (−1.23, −0.56)	−1.12 (−1.73, −0.68)	< 0.001
Section B. Time-weighted mean markers during the first year^*3*^					
Time-weighted mean eGFR (mL/min/1.73 m^2^)	106.87 (96.43, 117.82)	107.50 (97.64, 118.40)	99.97 (82.66, 116.70)	97.15 (87.43, 109.73)	< 0.001
Time-weighted mean CD4 (cells/µL)	299.49 (180.20, 442.00)	306.15 (185.95, 447.91)	161.00 (76.28, 274.20)	256.61 (148.68, 354.27)	< 0.001
Time-weighted mean combined eGFR-CD4 index	−1.69 (−2.29, −1.18)	−1.72 (−2.32, −1.20)	−1.07 (−1.55, −0.76)	−1.47 (−1.88, −1.01)	< 0.001

A combined eGFR-CD4 index = −0.004062488×eGFR-0.004197109×CD4

^*1*^Data are presented as median (Q1, Q3) or n (%). Missing data: marital status (*n* = 2), body mass index (*n* = 237), transmission route (*n* = 5), initial ART regimen (*n* = 1), and time to ART initiation (*n* = 2)

^*2*^Continuous variables were compared using the Kruskal–Wallis rank-sum test. Categorical variables were compared using Pearson’s chi-square test or Fisher’s exact test, as appropriate

^*3*^Participants who did not survive to the 1-year landmark (n = 398) were excluded

### Time-dependent Cox regression results

Time-dependent Cox proportional hazards models were fitted to evaluate the associations of eGFR, CD4 count, and the combined eGFR-CD4 index with all-cause, HIV-related, and non-HIV-related mortality. After adjustment for sex, age, occupational status, education level, marital status, BMI, disease stage, diagnostic delay, transmission route, initial ART regimen, and time to ART initiation, eGFR was not significantly associated with either all-cause mortality or non-HIV-related mortality (both *P* > 0.05). In contrast, CD4 count was independently associated with all-cause mortality, HIV-related mortality, and non-HIV-related mortality (aHR = 0.996, 95%CI: 0.995–0.997, *P* < 0.001; aHR = 0.99, 95%CI: 0.98–0.99, *P* < 0.001; aHR = 0.997, 95%CI: 0.995–0.998, *P* < 0.001, respectively).

For the combined eGFR-CD4 index (Index = −0.004062488 × eGFR − 0.004197109 × CD4), each 1-unit increase was associated with a 172% higher risk of all-cause mortality, a 1200% higher risk of HIV-related mortality, and a 132% higher risk of non-HIV-related mortality (aHR: 2.72, 95%CI: 2.05–3.61, *P* < 0.001; aHR: 13.00, 95%CI: 4.73–35.77, *P* < 0.001; aHR: 2.32, 95%CI: 1.75–3.08, *P* < 0.001, respectively). When additionally scaled to 1 SD, the associations remained robust for all-cause, HIV-related, and non–HIV-related mortality (aHR per 1 SD, 4.12 [95%CI, 2.76–6.15], *P* < 0.001; 37.86 [95%CI, 9.03–158.80], *P* < 0.001; and 3.30 [95%CI, 2.20–4.93], *P* < 0.001, respectively). Sensitivity analyses yielded similar results (Supplementary Table S2). The association between CBI and mortality was materially unchanged when CBI was scaled per IQR, when CBI values were restricted to the 1st–99th percentiles, and when deaths within the first 12 months of follow-up were excluded.

When categorized into quartiles, participants in the highest quartile (Q4) had a significantly higher risk of all-cause mortality (aHR: 3.12, 95%CI: 2.02–4.83, *P* < 0.001), HIV-related mortality (aHR: 11.13, 95%CI: 1.20–103.00, *P* = 0.034), and non-HIV-related mortality (aHR: 2.70, 95%CI: 1.71–4.25, *P* < 0.001) compared with those in the lowest quartile (Q1) (Table 2). Supplementary Figure S1 shows Kaplan-Meier survival curves for 12-month landmark all-cause, HIV-related, and non-HIV-related mortality, stratified by quartiles (Q1–Q4) of the combined eGFR-CD4 index. Across all three panels, survival decreased from Q1 to Q4, with significant differences across quartiles (log-rank test, all *P* < 0.001), consistent with the Cox regression results. The CIF curves also demonstrated graded risk separation across CBI quartiles, with higher quartiles showing a higher cumulative incidence of both HIV-related and non-HIV-related mortality after the 1-year landmark (Supplementary Figure S2).

**Table 2:** Time-dependent Cox regression analysis of eGFR, CD4, and their combined index in relation to all-cause, HIV-related, and non-HIV-related mortality

Index	Model 1		Model 2		Model 3	
	HR (95%CI)	*P*	HR (95%CI)	*P*	HR (95%CI)	*P*
All-cause mortality						
eGFR	0.97 (0.97, 0.98)	< 0.001	0.996 (0.989, 1.003)	0.220	0.997 (0.990, 1.004)	0.366
Q2 vs. Q1	0.50 (0.39, 0.64)	< 0.001	0.83 (0.63, 1.10)	0.202	0.86 (0.65, 1.14)	0.290
Q3 vs. Q1	0.41 (0.32, 0.52)	< 0.001	1.24 (0.87, 1.76)	0.231	1.27 (0.90, 1.79)	0.177
Q4 vs. Q1	0.25 (0.18, 0.33)	< 0.001	1.42 (0.87, 2.32)	0.160	1.57 (0.97, 2.54)	0.066
CD4	0.996 (0.995, 0.996)	< 0.001	0.996 (0.995, 0.997)	< 0.001	0.996 (0.995, 0.997)	< 0.001
Q2 vs. Q1	0.39 (0.31, 0.49)	< 0.001	0.41 (0.32, 0.53)	< 0.001	0.43 (0.33, 0.56)	< 0.001
Q3 vs. Q1	0.24 (0.18, 0.33)	< 0.001	0.35 (0.25, 0.48)	< 0.001	0.36 (0.25, 0.51)	< 0.001
Q4 vs. Q1	0.17 (0.12, 0.24)	< 0.001	0.30 (0.20, 0.43)	< 0.001	0.31 (0.20, 0.48)	< 0.001
Combined index^1^	3.21 (2.66, 3.87)	< 0.001	2.52 (2.02, 3.14)	< 0.001	2.72 (2.05, 3.61)	< 0.001
Combined index per SD^2^	5.22 (4.00, 6.81)	< 0.001	3.71 (2.72, 5.06)	< 0.001	4.12 (2.76, 6.15)	< 0.001
Q2 vs. Q1	1.35 (0.85, 2.14)	0.201	1.08 (0.68, 1.72)	0.736	1.07 (0.67, 1.70)	0.781
Q3 vs. Q1	2.32 (1.54, 3.51)	< 0.001	1.38 (0.91, 2.11)	0.128	1.36 (0.88, 2.10)	0.171
Q4 vs. Q1	6.53 (4.50, 9.50)	< 0.001	3.33 (2.26, 4.91)	< 0.001	3.12 (2.02, 4.83)	< 0.001
HIV-related mortality						
eGFR	0.991 (0.977, 1.004)	0.179	1.010 (1.003, 1.018)	0.004	1.012 (1.005, 1.018)	< 0.001
Q2 vs. Q1	0.79 (0.41, 1.52)	0.474	1.65 (0.75, 3.62)	0.215	1.84 (0.82, 4.14)	0.140
Q3 vs. Q1	0.66 (0.33, 1.30)	0.230	2.37 (0.84, 6.64)	0.102	2.48 (0.89, 6.86)	0.081
Q4 vs. Q1	0.70 (0.36, 1.33)	0.273	4.64 (1.18, 18.18)	0.028	5.85 (1.56, 21.97)	0.009
CD4	0.99 (0.99, 0.99)	< 0.001	0.99 (0.98, 0.99)	< 0.001	0.99 (0.98, 0.99)	< 0.001
Q2 vs. Q1	0.11 (0.04, 0.26)	< 0.001	0.08 (0.02, 0.24)	< 0.001	0.10 (0.03, 0.33)	< 0.001
Q3 vs. Q1	0.10 (0.04, 0.28)	< 0.001	0.07 (0.02, 0.30)	< 0.001	0.11 (0.03, 0.49)	0.004
Q4 vs. Q1	0.03 (0.00, 0.20)	< 0.001	0.05 (0.01, 0.32)	0.002	0.09 (0.01, 0.81)	0.033
Combined index^1^	10.27 (5.57, 18.91)	< 0.001	14.93 (6.67, 33.45)	< 0.001	13.00 (4.73, 35.77)	< 0.001
Combined index per SD^2^	27.08 (11.40, 64.35)	< 0.001	46.06 (14.69, 144.40)	< 0.001	37.86 (9.03, 158.80)	< 0.001
Q2 vs. Q1	3.62 (0.41, 32.15)	0.248	1.55 (0.14, 17.02)	0.720	1.29 (0.11, 15.26)	0.842
Q3 vs. Q1	2.30 (0.24, 21.92)	0.468	1.10 (0.10, 11.49)	0.939	0.76 (0.06, 8.96)	0.830
Q4 vs. Q1	38.80 (5.50, 273.74)	< 0.001	21.23 (3.12, 144.70)	0.002	11.13 (1.20, 103.00)	0.034
non-HIV related mortality						
eGFR	0.97 (0.97, 0.98)	< 0.001	0.9926 (0.9850, 1.0003)	0.060	0.994 (0.986, 1.001)	0.102
Q2 vs. Q1	0.47 (0.36, 0.61)	< 0.001	0.75 (0.56, 1.01)	0.062	0.77 (0.57, 1.04)	0.085
Q3 vs. Q1	0.38 (0.29, 0.50)	< 0.001	1.12 (0.77, 1.63)	0.548	1.14 (0.79, 1.65)	0.473
Q4 vs. Q1	0.19 (0.14, 0.27)	< 0.001	1.18 (0.70, 1.99)	0.529	1.29 (0.77, 2.15)	0.337
CD4	0.996 (0.995, 0.997)	< 0.001	0.997 (0.996, 0.998)	< 0.001	0.997 (0.995, 0.998)	< 0.001
Q2 vs. Q1	0.46 (0.36, 0.60)	< 0.001	0.50 (0.38, 0.65)	< 0.001	0.50 (0.38, 0.66)	< 0.001
Q3 vs. Q1	0.28 (0.20, 0.39)	< 0.001	0.42 (0.30, 0.59)	< 0.001	0.41 (0.28, 0.61)	< 0.001
Q4 vs. Q1	0.20 (0.14, 0.30)	< 0.001	0.37 (0.25, 0.54)	< 0.001	0.36 (0.23, 0.57)	< 0.001
Combined index^1^	2.78 (2.30, 3.37)	< 0.001	2.12 (1.70, 2.64)	< 0.001	2.32 (1.75, 3.08)	< 0.001
Combined index per SD^2^	4.26 (3.24, 5.59)	< 0.001	2.90 (2.12, 3.96)	< 0.001	3.30 (2.20, 4.93)	< 0.001
Q2 vs. Q1	1.28 (0.79, 2.05)	0.317	1.06 (0.66, 1.70)	0.806	1.07 (0.66, 1.72)	0.787
Q3 vs. Q1	2.34 (1.54, 3.56)	< 0.001	1.38 (0.90, 2.12)	0.139	1.40 (0.89, 2.18)	0.142
Q4 vs. Q1	5.38 (3.66, 7.92)	< 0.001	2.70 (1.80, 4.03)	< 0.001	2.70 (1.71, 4.25)	< 0.001

^1^Combined index =−0.004062488×eGFR−0.004197109×CD4, higher index values indicate lower eGFR and CD4

^2^Per SD indicates hazard ratios per 1-SD increase in the combined index

Model 1: unadjusted

Model 2: adjusted for sex, age, occupational status, education level, marital status, and BMI

Model 3: Model 2 plus disease stage, diagnostic delay, transmission route, initial ART regimen, and time to ART initiation

### Results of RCS analysis

**Fig. 2:**
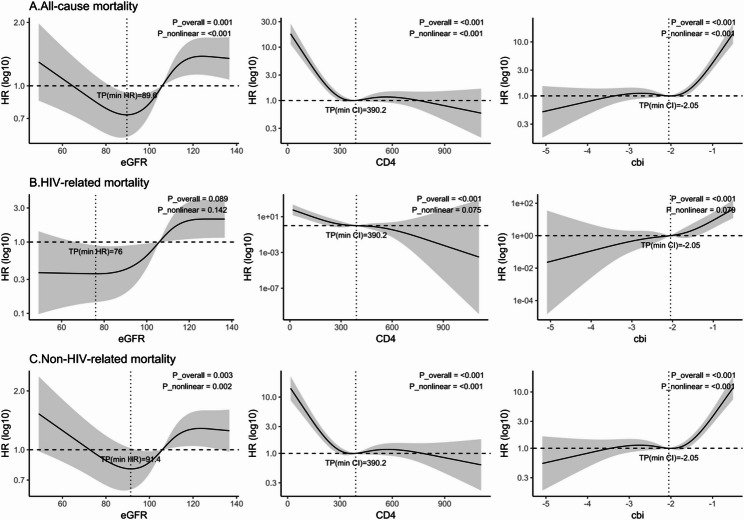
Restricted cubic spline associations of time-updated eGFR, CD4, and combined eGFR-CD4 index with mortality. Curves are shown within the 1st-99th percentiles; HRs are centered at the median (HR = 1) and plotted on a log10 scale

RCS models demonstrated nonlinear dose-response patterns for eGFR, CD4, and the combined eGFR-CD4 index across outcomes (Fig. 2). For all-cause mortality, eGFR was nonlinearly associated with risk (P_overall_ = 0.001; P_nonlinear_ < 0.001), with lowest hazard in the mid-range and higher hazard at both lower and higher values. CD4 exhibited an inverse, nonlinear association (P_overall_ < 0.001; P_nonlinear_ < 0.001), with elevated risk at low CD4 levels and a flatter gradient at higher levels. The combined eGFR-CD4 index was nonlinearly associated with all-cause mortality (P_overall_ < 0.001; P_nonlinear_ < 0.001), with risk increasing toward higher index values.

For HIV-related mortality, eGFR was not significantly associated (P_overall_ = 0.089; P_nonlinear_ = 0.142). CD4 remained strongly associated (P_overall_ < 0.001) with no clear evidence of nonlinearity (P_nonlinear_ = 0.075). The combined index was similarly associated (P_overall_ < 0.001) with borderline nonlinearity (P_nonlinear_ = 0.079). For non-HIV-related mortality, eGFR (P_overall_ = 0.003; P_nonlinear_ = 0.002), CD4 (P_overall_ < 0.001; P_nonlinear_ < 0.001), and combined index (P_overall_ < 0.001; P_nonlinear_ < 0.001) were all significantly and nonlinearly associated with risk. Confidence intervals widened at the distribution tails, consistent with fewer observations at extreme biomarker values.

### Results of time-dependent ROC analysis

**Fig. 3:**
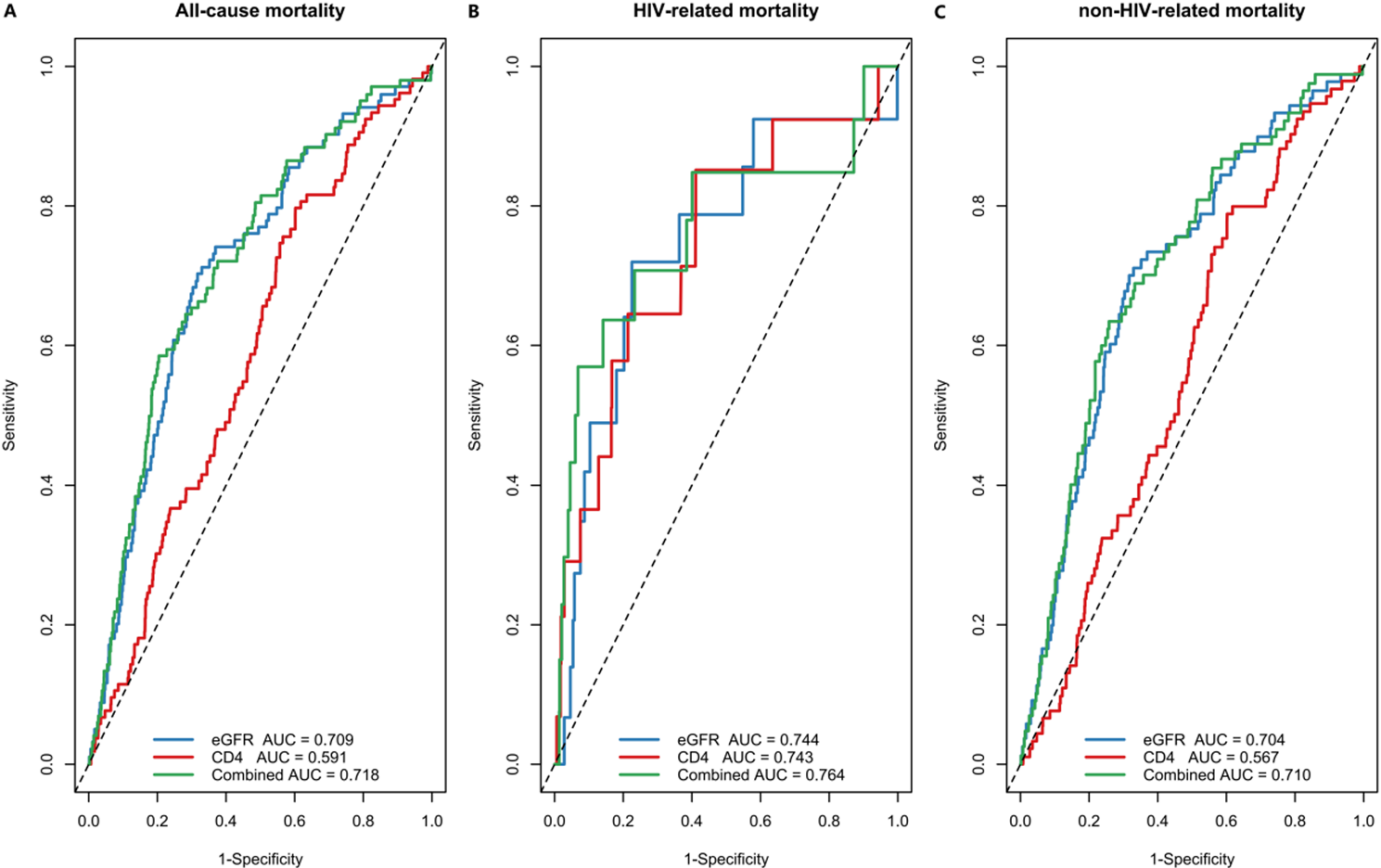
Time-dependent ROC curves assessing the predictive performance of eGFR, CD4, and their combined score at 3 years after the 1-year landmark. **A** All-cause mortality; **B** HIV-related mortality; **C** non-HIV-related mortality

Time-dependent ROC analyses were performed to compare the discrimination of eGFR, CD4, and the combined eGFR-CD4 score for mortality outcomes. At 3 years after the 1-year landmark, the combined score showed the highest apparent AUCs for all-cause, HIV-related, and non-HIV-related mortality, with corresponding AUCs of 0.718, 0.764, and 0.710, respectively (Fig. 3). A similar pattern was observed for the apparent 5-year AUCs (Supplementary Figure S3).

To assess the extent of optimism in these apparent discrimination estimates, we then performed bootstrap internal validation. In bootstrap internal validation with 200 resamples, the optimism-corrected 3-year AUCs for all-cause mortality were 0.696 for eGFR alone, 0.598 for CD4 alone, and 0.709 for the combined model; the corresponding 5-year AUCs were 0.697, 0.574, and 0.701, respectively. For HIV-related mortality, the optimism-corrected 3-year AUCs were 0.701 for eGFR alone, 0.745 for CD4 alone, and 0.739 for the combined model; the corresponding 5-year AUCs were 0.596, 0.707, and 0.695, respectively. For non-HIV-related mortality, the optimism-corrected 3-year AUCs were 0.692 for eGFR alone, 0.577 for CD4 alone, and 0.702 for the combined model; the corresponding 5-year AUCs were 0.707, 0.556, and 0.709, respectively. Overall, optimism was small across analyses. The combined model showed a modest but consistent discriminatory advantage for all-cause and non-HIV-related mortality, whereas for HIV-related mortality the highest optimism-corrected discrimination was observed for CD4 alone.

### Subgroup analysis

Subgroup analyses were conducted after stratifying participants by sex, age, occupational status, education level, marital status, BMI, disease stage, diagnostic delay, transmission route, initial ART regimen, and time to ART initiation. The combined eGFR-CD4 index remained significantly associated with all-cause mortality across most strata (all *P* < 0.05), with the exception of the subgroups of females, participants with stable employment, those with a diagnostic delay of 3–12 months, and those initiating ART within 1–3 months, in which the association did not reach statistical significance. Significant effect modification was observed for age and disease stage (interaction *P* = 0.043 and 0.007, respectively) (Supplementary Figure S4). Results were consistent when the index was modeled per SD increase (Supplementary Figure S5).

## Discussion

In this cohort study of PWH, time-updated CD4 was consistently associated with all-cause, HIV-related, and non-HIV-related mortality. For all-cause and non-HIV-related mortality, eGFR demonstrated a nonlinear association in spline models, whereas its linear association was attenuated after full adjustment. The CBI was associated with all three mortality outcomes, and in the primary landmark analyses showed higher apparent discrimination than the single-biomarker models. In a 12-month landmark analysis, higher CBI quartiles stratified subsequent risk, including higher cumulative incidence of both HIV-related and non-HIV-related death. In bootstrap internal validation, the combined model retained a modest discriminatory advantage for all-cause and non-HIV-related mortality, whereas for HIV-related mortality CD4 alone had the highest optimism-corrected discrimination. These findings suggest that the apparent performance of the combined model was not materially driven by optimism for all-cause and non-HIV-related mortality, but that its added value over CD4 for HIV-related mortality was limited. Given the observational design and the potential for residual confounding, these findings should be interpreted as associative rather than predictive.

The observed CD4 associations align with longitudinal evidence showing a strong, graded relationship between immune status and subsequent mortality, with excess risk concentrated among individuals with low CD4 counts. In our RCS analyses, risk increased most sharply at lower CD4 levels and plateaued at higher counts, consistent with prior cohort observations that the clinical impact of immune suppression is greatest below conventional thresholds (e.g., 200–350 cells/µL) [20–23]. The internal validation results for HIV-related mortality further support the central prognostic importance of immune status, as CD4 alone achieved the highest optimism-corrected AUCs at both 3 and 5 years. Although CD8 data were available, the present study was designed to focus on a parsimonious renal-immune index based on two routinely used markers, eGFR and CD4. CD8 count and the CD4/CD8 ratio were therefore not incorporated into the primary analyses. Future studies should evaluate whether these immune markers provide additional prognostic value beyond CD4 and the combined eGFR-CD4 index.

Prior studies have linked impaired kidney function to higher mortality among PWH, but reported thresholds and effect sizes vary across settings and analytic approaches [24, 25]. In our cohort, the nonlinear association between eGFR and all-cause and non-HIV-related mortality suggests that risk may not change at a constant rate across the eGFR distribution, which may partly explain why a single linear term attenuated after full adjustment [26]. Because eGFR physiologically declines with age, age-related renal decline may also have contributed to the observed associations despite multivariable adjustment. For HIV-related mortality, we observed a positive association with higher creatinine-based eGFR, but this counterintuitive finding should be interpreted cautiously given the limited number of HIV-related deaths and the resulting instability of cause-specific estimates. This pattern may reflect non-GFR determinants of Scr in advanced HIV disease (e.g., low muscle mass, wasting, and systemic inflammation), which can spuriously inflate eGFR estimates [27, 28]. This finding underscores limitations of relying solely on creatinine-based eGFR for HIV-related risk assessment and supports consideration of complementary kidney or frailty-related markers in future work.

CBI combines 2 routinely collected measures that reflect distinct biologic domains. CD4 captures immune status and HIV disease severity, whereas eGFR captures kidney function and broader comorbidity burden. The combined index may therefore capture prognostic information not fully represented by either marker alone. This rationale is consistent with established multimarker prognostic tools in HIV care, such as the VACS Index framework, which integrates HIV-related and non-HIV-related biomarkers to improve mortality risk discrimination [12, 29–31]. In contrast to more parameter-rich scores, CBI uses only 2 widely available laboratory measures, which may support pragmatic summary assessment of renal-immune status in research and surveillance settings. However, the incremental gain of the combined model over eGFR alone for all-cause and non-HIV-related mortality was modest, and the combined model did not outperform CD4 for HIV-related mortality after optimism correction. Because the CBI is mathematically derived from eGFR and CD4, it should not be interpreted as an independent biomarker separate from its component measures. Thus, the discriminatory value of the CBI should be interpreted as exploratory rather than definitive.

We evaluated HIV-related and non-HIV-related mortality as separate outcomes. The results suggest that the renal-immune profile may be more informative for all-cause and non-HIV-related mortality than for HIV-related mortality, for which CD4 alone remained the strongest discriminator after internal validation. The competing-risk cumulative incidence curves (Supplementary Figure S2) further support risk separation across CBI quartiles while appropriately accounting for competing causes of death [32, 33]. For eGFR, associations were more evident for non-HIV-related mortality in spline analyses, consistent with evidence that kidney dysfunction clusters with cardiometabolic and other non-HIV comorbidities that account for a growing share of deaths in the ART era [3, 34]. However, cause-of-death misclassification and limited event counts in some categories could have attenuated or destabilized cause-specific estimates [35].

Associations between CBI and all-cause mortality were generally consistent across prespecified subgroups, with evidence of effect modification by age and disease stage. These subgroup findings warrant cautious interpretation because several strata had fewer events, limiting statistical power and widening confidence intervals [36]. Nevertheless, the observed pattern is biologically plausible given heterogeneity in multimorbidity burden and baseline HIV severity across age groups and disease stages [37, 38].

Taken together, these findings support continued joint consideration of immune status and kidney function in longitudinal risk assessment, but they do not establish the CBI as a validated prognostic tool for routine clinical use. Rather, the index may be most useful as a simple exploratory summary of renal-immune status, particularly for all-cause and non-HIV-related mortality, pending external validation. Current guidance emphasizes continued clinical monitoring of immune status and organ function during ART, and renal function remains important for antiretroviral selection and dosing, including avoidance of potentially nephrotoxic exposures in higher-risk individuals [39–41]. Accordingly, our findings should be viewed as supporting further evaluation of a parsimonious renal-immune index rather than immediate clinical implementation.

This study has limitations. Residual confounding remains an important consideration, particularly because viral suppression status is highly relevant to both CD4 trajectories and cause-specific mortality. Unavailable or incompletely measured factors, including viral load, ART adherence, hepatitis B or C coinfection, proteinuria, and comorbidity burden, may therefore have influenced biomarker trajectories and mortality outcomes. The requirement for at least one paired Scr and CD4 measurement, and for repeated measurements in the time-updated analyses, may have introduced selection bias by preferentially including individuals with better healthcare access and more complete follow-up, thereby limiting the representativeness of the analytic cohort.

Biomarker updating using interval carry-forward was pragmatic for this surveillance-based cohort, but it assumes relative stability between observation times and may not capture rapid biological changes; more intensive clinical monitoring in some participants may also have introduced informative observation bias. Although bootstrap internal validation was performed, the CBI was still derived and evaluated within the same underlying cohort, and external validation in an independent population was not available. Formal validation of concordance and completeness between CRIMS and the Taizhou cause-of-death surveillance system was also unavailable, and some under-ascertainment or misclassification of HIV-related versus non-HIV-related deaths may therefore remain possible, particularly given the limited number of HIV-related events. Finally, this single-city cohort may further limit generalizability; external validation in other settings is warranted.

## Conclusions

In conclusion, in this real-world cohort of PWH in Taizhou, Zhejiang, China, time-updated CD4 and a combined eGFR-CD4 index were associated with all-cause, HIV-related, and non-HIV-related mortality. The combined model showed modestly higher discrimination for all-cause and non-HIV-related mortality, but not for HIV-related mortality, for which CD4 alone performed best. These findings support further external validation of a simple renal-immune index as an exploratory tool for longitudinal risk stratification in the ART era.

## Supplementary Information

Supplementary Material.

## Data Availability

The datasets used and/or analyzed during the current study are not publicly available due to privacy and ethical restrictions, but are available from the corresponding author on reasonable request.

## Abbreviations

ART: Antiretroviral therapy
CRIMS: China HIV/AIDS Comprehensive Response Information Management System
CBI: Combined eGFR-CD4 index
CIFs: Cumulative incidence functions
CIs: Confidence intervals
eGFR: Estimated glomerular filtration rate
HRs: Hazard ratios
PWH: People with HIV
ROC: Receiver operating characteristic
RCS: Restricted cubic splines
Scr: Serum creatinine
VACS: Veterans Aging Cohort Study
