# Supplementary material for: A combined eGFR-CD4 index and mortality risk among people with HIV: a retrospective cohort study in Taizhou city, Zhejiang province, China
Source: AIDS Res Ther. 2026 Jun 7;23:76. doi: 10.1186/s12981-026-00894-1 (PMC13471255; doi:10.1186/s12981-026-00894-1)
Supplement: Supplementary file 1 [file 12981_2026_894_MOESM1_ESM.docx]

**Supplementary materials**

**Supplementary Methods.** Additional Methodological Details

**Table S1.** The STROBE reporting checklist

**Table S2.** Sensitivity Analyses of the Association Between the Combined eGFR-CD4 Index and Mortality

**Figure S1**. Kaplan-Meier survival curves for 12-month landmark mortality from the first measurement among PWH by quartiles (Q1-Q4) of the combined eGFR-CD4 index: (A) all-cause; (B) HIV-related; (C) non-HIV-related mortality.

**Figure S2**. Cumulative incidence functions for HIV-related and non-HIV-related mortality by CBI quartiles (Q1–Q4)

**Figure S3**. Time-dependent ROC curves assessing the predictive performance of eGFR, CD4, and their combined score at 5 years after the 1-year landmark

**Figure S4**. Subgroup analyses of the association between combined eGFR-CD4 index (per 1-unit increase) and all-cause mortality

**Figure S5**. Subgroup analyses of the association between combined eGFR-CD4 index (per 1-SD increase) and all-cause mortality

**Supplementary Methods**. Additional Methodological Details

**Cause-of-death classification**

HIV-related deaths were defined as deaths reported or verified as AIDS or AIDS-defining opportunistic infections in the detailed cause-of-death field. In addition, deaths were classified as HIV-related if the underlying cause of death recorded using the International Statistical Classification of Diseases and Related Health Problems, Tenth Revision (ICD-10) codes indicated AIDS-related conditions, including AIDS-related sepsis, pulmonary infection/pneumonia, infectious shock, tuberculosis, or meningitis. Deaths were also classified as HIV-related when AIDS was not explicitly mentioned in the detailed, immediate, or underlying cause-of-death fields but the recorded diagnosis was consistent with AIDS-defining conditions, such as cryptococcosis, Pneumocystis jirovecii pneumonia, non-Hodgkin lymphoma, or pneumocystis pneumonia. All other deaths not attributed to AIDS—primarily deaths due to chronic noncommunicable diseases—were classified as non-HIV-related deaths. Cause-of-death information was obtained through linkage between CRIMS and the Taizhou cause-of-death surveillance system using unique identifiers. Formal validation of concordance and completeness between the two linked systems was not available in the present study.

**Start–stop data structure and updating rules**

We constructed a counting-process (start–stop) dataset using the *tmerge* function in the R *survival* package. Follow-up for each participant was partitioned into consecutive intervals defined by laboratory measurement dates. Baseline (time zero) was defined as the date of the first paired eGFR and CD4 observation. In the routine surveillance and clinical care setting, serum creatinine and CD4 were treated as paired observations for longitudinal analysis, although laboratory testing or reporting could occur on different dates. Time-dependent covariates were updated at each new paired observation; within each interval, the most recent observed values of eGFR and CD4 were carried forward until the next available paired observation, death, or censoring (last observation carried forward). This approach assumes relative stability of biomarker values between observation times and may not capture rapid biological changes.

**First-year time-weighted mean biomarkers**

To summarize biomarker exposure during the first year after baseline, we computed time-weighted mean eGFR, CD4, and CBI for each participant. Let intervals within the first year be indexed by i, with duration Δtᵢ, and biomarker value Xᵢ assumed constant within each interval under last observation carried forward. The first-year time-weighted mean was calculated as:

$${\overline{\text{X}}}_{\text{0−12}\text{mo}}\text{=}\frac{\sum_{\text{i}} \text{X}_{\text{i}}\text{∙△}\text{t}_{\text{i}}}{\sum_{\text{i}} \text{△}\text{t}_{\text{i}}}$$

The same approach was used for eGFR, CD4, and CBI. These first-year summaries were used for descriptive comparisons in Table 1 and to assign quartiles for the 12-month landmark Kaplan-Meier analyses. They were also used to generate landmark-based risk scores for time-dependent ROC analyses.

**Construction and standardization of the combined index**

CBI was computed for each follow-up interval as:

$$\text{CBI}\text{(}\text{t}\text{)=}\text{β}_{\text{eGFR}}\text{∙}\text{eGFR}\text{(}\text{t}\text{)+}\text{β}_{\text{CD}\text{4}}\text{∙}\text{CD}\text{4(}\text{t}\text{)}$$

with $\text{β}_{\text{eGFR}}$ = −0.004062488 and $\text{β}_{\text{CD}\text{4}}$ = −0.004197109, yielding:

$$\text{CBI}\text{(}\text{t}\text{)=}\text{ -0.004062488}\text{∙}\text{eGFR}\text{(}\text{t}\text{)+}\text{-0.004197109}\text{∙}\text{CD}\text{4(}\text{t}\text{)}$$

The coefficients used to define the CBI were estimated from the fully adjusted time-dependent Cox model in the same analytic cohort in which the index was subsequently evaluated. Therefore, the CBI should be interpreted as an exploratory composite marker rather than a pre-specified validated score.

For Cox models, CBI was standardized using z transformation based on the analytic sample:

$$\text{CBI}_{\text{z}}\text{=}\frac{\text{CBI}\text{−}\text{μ}}{\text{σ}}$$

where $\text{μ}$ and $\text{σ}$ denote the mean and SD of CBI in the analytic dataset. HRs were reported per 1-unit increase in CBI (original scale) and per 1-SD increase in standardized CBI (z score). Prespecified sensitivity analyses were performed to assess robustness: CBI was additionally scaled per interquartile range (IQR) increase; models were refit after restricting CBI to the 1st–99th percentiles to reduce the influence of extreme values; and participants who died within the first 12 months of follow-up were excluded to mitigate potential reverse causation.

**Outcomes**

For cause-specific hazard models, HIV-related deaths were treated as the event of interest and deaths from other causes were censored at the time of death; non-HIV-related deaths were handled analogously. Underlying cause of death was obtained from the cause-of-death surveillance system. Cumulative incidence functions (CIFs) were also estimated using the Aalen-Johansen method accounting for competing risks.

**Time-dependent ROC analysis (cause-specific censoring approach)**

Time-dependent ROC curves were estimated using the *timeROC* package with marginal weighting. Risk scores were derived from Cox models fit from the 12-month landmark onward for eGFR-only, CD4-only, and combined models. For HIV-related mortality, time-to-event was defined as time from the landmark to HIV-related death; participants who died from non-HIV-related causes were censored at their time of death, and HIV-related deaths were coded as events. Non-HIV-related mortality was handled analogously. AUCs were estimated at 3 years after the landmark; 5-year AUCs were examined in secondary analyses.

Bootstrap internal validation was performed within the 12-month landmark framework to assess potential optimism in model discrimination. Using 200 participant-level bootstrap resamples, each Cox model was refitted in the bootstrap sample and then evaluated both in the bootstrap sample and in the original dataset. Optimism was calculated as the difference between the bootstrap apparent AUC and the bootstrap test AUC. The mean optimism across bootstrap samples was subtracted from the apparent AUC in the original dataset to obtain the optimism-corrected AUC. For cause-specific outcomes, deaths from other causes were treated as censoring events.

**Restricted cubic splines**

RCS terms with 4 knots were fitted using Cox models to flexibly model biomarker–outcome relationships. HRs were centered at the median of each biomarker (HR = 1) and plotted on a log10 scale. Plots were restricted to the 1st–99th percentiles to reduce instability from extreme values. Overall and nonlinear effects were tested using Wald statistics.

**Software**

All analyses were performed in R (version 4.3.1), primarily using the *survival*, *timeROC*, and *rms* packages.

**Table S1.** The STROBE reporting checklist

|  | Item Description | Location (or reason for not reporting) |
| --- | --- | --- |
| **Title and abstract** |  |  |
| [1a. Indicate the study’s design](https:/resources.equator-network.org/reporting-guidelines/strobe/items/title-abstract-indicate-study-design.html?utm_source=strobe&utm_medium=checklist&utm_campaign=1_1) | Indicate the study’s design with a commonly used term in the title or the abstract. | Title and Abstract |
| [1b. Abstract](https:/resources.equator-network.org/reporting-guidelines/strobe/items/abstract.html?utm_source=strobe&utm_medium=checklist&utm_campaign=1_1) | Provide in the abstract an informative and balanced summary of what was done and what was found. | Abstract |
| **Introduction** |  |  |
| [2. Background / rationale](https:/resources.equator-network.org/reporting-guidelines/strobe/items/background-rationale.html?utm_source=strobe&utm_medium=checklist&utm_campaign=1_1) | Explain the scientific background and rationale for the investigation being reported. | Introduction; paragraph 1-3 |
| [3. Objectives](https:/resources.equator-network.org/reporting-guidelines/strobe/items/objectives.html?utm_source=strobe&utm_medium=checklist&utm_campaign=1_1) | State specific objectives, including any prespecified hypotheses. | Introduction; paragraph 4 |
| **Methods** |  |  |
| [4. Study design](https:/resources.equator-network.org/reporting-guidelines/strobe/items/study-design.html?utm_source=strobe&utm_medium=checklist&utm_campaign=1_1) | Present key elements of study design early in the paper. | Methods, Research design and study population; paragraphs 1 |
| [5. Setting](https:/resources.equator-network.org/reporting-guidelines/strobe/items/setting.html?utm_source=strobe&utm_medium=checklist&utm_campaign=1_1) | Describe the setting, locations, and relevant dates, including periods of recruitment, exposure, follow-up, and data collection. | Methods, Research design and study population; paragraphs 1&2 |
| [6a. Eligibility criteria](https:/resources.equator-network.org/reporting-guidelines/strobe/items/eligibility-criteria.html?utm_source=strobe&utm_medium=checklist&utm_campaign=1_1) | **Cohort study:** Give the eligibility criteria, and the sources and methods of selection of participants. Describe methods of follow-up. **Case-control study:** Give the eligibility criteria, and the sources and methods of case ascertainment and control selection. Give the rationale for the choice of cases and controls. **Cross-sectional study:** Give the eligibility criteria, and the sources and methods of selection of participants. | Methods, Research design and study population; paragraphs 2  Figure 1 |
| [6b. Matching criteria](https:/resources.equator-network.org/reporting-guidelines/strobe/items/matching-criteria.html?utm_source=strobe&utm_medium=checklist&utm_campaign=1_1) | **Cohort study:** For matched studies, give matching criteria and number of exposed and unexposed. **Case-control study:** For matched studies, give matching criteria and the number of controls per case. | Not applicable (study was not a matched cohort design) |
| [7. Variables](https:/resources.equator-network.org/reporting-guidelines/strobe/items/variables.html?utm_source=strobe&utm_medium=checklist&utm_campaign=1_1) | Clearly define all outcomes, exposures, predictors, potential confounders, and effect modifiers. Give diagnostic criteria, if applicable. | Methods, Time-varying biomarkers and combined eGFR-CD4 index & Outcomes |
| [8. Data sources / measurement](https:/resources.equator-network.org/reporting-guidelines/strobe/items/data-sources-measurement.html?utm_source=strobe&utm_medium=checklist&utm_campaign=1_1) | For each variable of interest give sources of data and details of methods of assessment (measurement). Describe comparability of assessment methods if there is more than one group. | Methods, Research design and study population; paragraphs 1 |
| [9. Bias](https:/resources.equator-network.org/reporting-guidelines/strobe/items/bias.html?utm_source=strobe&utm_medium=checklist&utm_campaign=1_1) | Describe any efforts to address potential sources of bias. | Methods, Time-dependent Cox regression |
| [10. Study size](https:/resources.equator-network.org/reporting-guidelines/strobe/items/study-size.html?utm_source=strobe&utm_medium=checklist&utm_campaign=1_1) | Explain how the study size was arrived at. | Methods, Research design and study population; paragraphs 2  Figure 1 |
| [11. Quantitative variables](https:/resources.equator-network.org/reporting-guidelines/strobe/items/quantitative-variables.html?utm_source=strobe&utm_medium=checklist&utm_campaign=1_1) | Explain how quantitative variables were handled in the analyses. If applicable, describe which groupings were chosen, and why. | Table 1 |
| [12a. Statistical methods](https:/resources.equator-network.org/reporting-guidelines/strobe/items/statistical-methods-description.html?utm_source=strobe&utm_medium=checklist&utm_campaign=1_1) | Describe all statistical methods, including those used to control for confounding. | Methods, Time-dependent Cox regression&Kaplan–Meier curves&Time-dependent ROC curves&Restricted cubic spline analysis |
| [12b. Statistical methods – subgroups and interactions](https:/resources.equator-network.org/reporting-guidelines/strobe/items/statistical-methods-subgroups-interactions.html?utm_source=strobe&utm_medium=checklist&utm_campaign=1_1) | Describe any methods used to examine subgroups and interactions. | Methods, Time-dependent Cox regression |
| [12c. Statistical methods – missing data](https:/resources.equator-network.org/reporting-guidelines/strobe/items/statistical-methods-missing-data.html?utm_source=strobe&utm_medium=checklist&utm_campaign=1_1) | Explain how missing data were addressed. | Methods, Time-dependent Cox regression |
| [12di. Statistical methods – loss to follow-up](https:/resources.equator-network.org/reporting-guidelines/strobe/items/statistical-methods-loss-to-follow-up.html?utm_source=strobe&utm_medium=checklist&utm_campaign=1_1) | **Cohort study:** If applicable, describe how loss to follow-up was addressed. | Methods, Outcomes |
| [12dii. Statistical methods – matching cases and controls](https:/resources.equator-network.org/reporting-guidelines/strobe/items/statistical-methods-matching-cases-controls.html?utm_source=strobe&utm_medium=checklist&utm_campaign=1_1) | **Case-control study:** If applicable, explain how matching of cases and controls was addressed. | Not applicable |
| [12diii. Statistical methods – sampling strategy](https:/resources.equator-network.org/reporting-guidelines/strobe/items/statistical-methods-analytical-methods-sampling-strategy.html?utm_source=strobe&utm_medium=checklist&utm_campaign=1_1) | **Cross-sectional study:** If applicable, describe analytical methods taking account of sampling strategy. | Not applicable |
| [12e. Statistical methods – sensitivity analyses](https:/resources.equator-network.org/reporting-guidelines/strobe/items/statistical-methods-sensitivity-analyses.html?utm_source=strobe&utm_medium=checklist&utm_campaign=1_1) | Describe any sensitivity analyses. | Methods, Time-dependent Cox regression |
| **Results** |  |  |
| [13a. Participant numbers](https:/resources.equator-network.org/reporting-guidelines/strobe/items/participants-numbers.html?utm_source=strobe&utm_medium=checklist&utm_campaign=1_1) | Report the numbers of individuals at each stage of the study—e.g., numbers potentially eligible, examined for eligibility, confirmed eligible, included in the study, completing follow-up, and analysed; Consider use of a flow diagram. | Results, Participant characteristics  Figure 1 |
| [13b. Participants – non-participation](https:/resources.equator-network.org/reporting-guidelines/strobe/items/participants-non-participation.html?utm_source=strobe&utm_medium=checklist&utm_campaign=1_1) | Give reasons for non-participation at each stage. | Methods, Research design and study population; paragraphs 2 |
| [13c. Participants – flow diagram](https:/resources.equator-network.org/reporting-guidelines/strobe/items/participants-flow-diagram.html?utm_source=strobe&utm_medium=checklist&utm_campaign=1_1) | Consider use of a flow diagram. | Figure 1 |
| [14a. Descriptive data – participant characteristics](https:/resources.equator-network.org/reporting-guidelines/strobe/items/descriptive-data-participant-characteristics.html?utm_source=strobe&utm_medium=checklist&utm_campaign=1_1) | Give characteristics of study participants (e.g., demographic, clinical, social) and information on exposures and potential confounders. Present the information in a table. | Results, Participant characteristics  Table 1 |
| [14b. Descriptive data – missing data](https:/resources.equator-network.org/reporting-guidelines/strobe/items/descriptive-data-missing-data.html?utm_source=strobe&utm_medium=checklist&utm_campaign=1_1) | Indicate the number of participants with missing data for each variable of interest. | Table 1 |
| [14c. Descriptive data – follow-up time](https:/resources.equator-network.org/reporting-guidelines/strobe/items/descriptive-data-follow-up-time.html?utm_source=strobe&utm_medium=checklist&utm_campaign=1_1) | **Cohort study:** Summarise follow-up time—e.g., average and total amount. | Results, Participant characteristics |
| [15. Outcome data](https:/resources.equator-network.org/reporting-guidelines/strobe/items/outcome-data.html?utm_source=strobe&utm_medium=checklist&utm_campaign=1_1) | **Cohort study:** Report numbers of outcome events or summary measures over time. **Case-control study:** Report numbers in each exposure category, or summary measures of exposure. **Cross-sectional study:** Report numbers of outcome events or summary measures. | Results, Participant characteristics |
| [16a. Main results](https:/resources.equator-network.org/reporting-guidelines/strobe/items/main-results.html?utm_source=strobe&utm_medium=checklist&utm_campaign=1_1) | Give unadjusted estimates and, if applicable, confounder-adjusted estimates and their precision (e.g., 95% confidence intervals). Make clear which confounders were adjusted for and why they were included. | Results, Time-dependent Cox regression results |
| [16b. Main results – category boundaries](https:/resources.equator-network.org/reporting-guidelines/strobe/items/main-results-category-boundaries.html?utm_source=strobe&utm_medium=checklist&utm_campaign=1_1) | Report category boundaries when continuous variables were categorised. | Table 1 |
| [16c. Main results – risk](https:/resources.equator-network.org/reporting-guidelines/strobe/items/main-results-risk.html?utm_source=strobe&utm_medium=checklist&utm_campaign=1_1) | If relevant, consider translating estimates of relative risk into absolute risk for a meaningful time period. | Not applicable (the exposure variables were modeled as time-updated continuous variables) |
| [17. Other analyses](https:/resources.equator-network.org/reporting-guidelines/strobe/items/other-analyses.html?utm_source=strobe&utm_medium=checklist&utm_campaign=1_1) | Report other analyses done—e.g., analyses of subgroups and interactions, and sensitivity analyses. | Results, Results of RCS analysis & Results of time-dependent ROC analysis & Subgroup analysis |
| **Discussion** |  |  |
| [18. Key results](https:/resources.equator-network.org/reporting-guidelines/strobe/items/key-results.html?utm_source=strobe&utm_medium=checklist&utm_campaign=1_1) | Summarise key results with reference to study objectives. | Discussion; paragraphs 1 |
| [19. Limitations](https:/resources.equator-network.org/reporting-guidelines/strobe/items/limitations.html?utm_source=strobe&utm_medium=checklist&utm_campaign=1_1) | Discuss limitations of the study, taking into account sources of potential bias or imprecision. Discuss both direction and magnitude of any potential bias. | Discussion; paragraphs 8 |
| [20. Interpretation](https:/resources.equator-network.org/reporting-guidelines/strobe/items/interpretation.html?utm_source=strobe&utm_medium=checklist&utm_campaign=1_1) | Give a cautious overall interpretation considering objectives, limitations, multiplicity of analyses, results from similar studies, and other relevant evidence. | Discussion; paragraphs 2-7 |
| [21. Generalisability](https:/resources.equator-network.org/reporting-guidelines/strobe/items/generalisability.html?utm_source=strobe&utm_medium=checklist&utm_campaign=1_1) | Discuss the generalisability (external validity) of the study results. | Discussion; paragraphs 9 |
| **Other information** |  |  |
| [22. Funding](https:/resources.equator-network.org/reporting-guidelines/strobe/items/funding.html?utm_source=strobe&utm_medium=checklist&utm_campaign=1_1) | Give the source of funding and the role of the funders for the present study and, if applicable, for the original study on which the present article is based. | Funding |

**Table S2**. Sensitivity Analyses of the Association Between the Combined eGFR-CD4 Index and Mortality

| Index | All-cause mortality |  |  | HIV-related mortality |  |  | Non–HIV-related mortality |  |
| --- | --- | --- | --- | --- | --- | --- | --- | --- |
|  | aHR (95%CI) | P |  | aHR (95%CI) | P |  | aHR (95%CI) | P |
| CBI per IQR increase | 3.68 (2.55, 5.31) | <0.001 |  | 28.21 (7.55, 105.30) | <0.001 |  | 2.99 (2.07, 4.33) | <0.001 |
| CBI, 1st-99th percentile restriction | 2.14 (1.64, 2.78) | <0.001 |  | 12.62 (3.77, 42.28) | <0.001 |  | 1.83 (1.41, 2.37) | <0.001 |
| Excluding deaths within the first 12 months of follow-up | 2.03 (1.52, 2.72) | <0.001 |  | 7.77 (2.51, 24.01) | <0.001 |  | 1.81 (1.35, 2.43) | <0.001 |

Primary Cox results (CBI per 1-SD and per 1-unit) are shown in Table 2; eTable 1 presents additional sensitivity analyses.

**Figure S1**. Kaplan-Meier survival curves for 12-month landmark mortality from the first measurement among PWH by quartiles (Q1-Q4) of the combined eGFR-CD4 index: (A) all-cause; (B) HIV-related; (C) non-HIV-related mortality.

**Figure S2**. Cumulative incidence functions for HIV-related and non-HIV-related mortality by CBI quartiles (Q1–Q4)

**Figure S3**. Time-dependent ROC curves assessing the predictive performance of eGFR, CD4, and their combined score at 5 years after the 1-year landmark. (A) All-cause mortality; (B) HIV-related mortality; (C) non-HIV-related mortality.

**Figure S4**. Subgroup analyses of the association between combined eGFR-CD4 index (per 1-unit increase) and all-cause mortality

**Figure S5**. Subgroup analyses of the association between combined eGFR-CD4 index (per 1-SD increase) and all-cause mortality
